# An Overview on Spray-Drying of Protein-Loaded Polymeric Nanoparticles for Dry Powder Inhalation

**DOI:** 10.3390/pharmaceutics12111032

**Published:** 2020-10-29

**Authors:** Tânia Marante, Cláudia Viegas, Inês Duarte, Ana S. Macedo, Pedro Fonte

**Affiliations:** 1 Center for Marine Sciences (CCMar), University of Algarve, Gambelas Campus, 8005-139 Faro, Portugal; taniamarante1993@gmail.com (T.M.); viegas.claudiasofia@gmail.com (C.V.); 2 Department of Chemistry and Pharmacy, Faculty of Sciences and Technology, University of Algarve, Gambelas Campus, 8005-139 Faro, Portugal; 3 Institute for Bioengineering and Biosciences (iBB), Department of Bioengineering, Instituto Superior Técnico, Universidade de Lisboa, 1049-001 Lisboa, Portugal; ines.v.duarte@tecnico.ulisboa.pt; 4 LAQV, REQUIMTE, Department of Chemical Sciences–Applied Chemistry Lab, Faculty of Pharmacy, University of Porto, Rua Jorge de Viterbo Ferreira 228, 4050-313 Porto, Portugal; p5514@ulusofona.pt

**Keywords:** protein delivery, polymeric nanoparticle, lung, spray-drying, inhalation, dry powder, pulmonary delivery, atomization, particle deposition, bronchi

## Abstract

The delivery of therapeutic proteins remains a challenge, despite recent technological advances. While the delivery of proteins to the lungs is the gold standard for topical and systemic therapy through the lungs, the issue still exists. While pulmonary delivery is highly attractive due to its non-invasive nature, large surface area, possibility of topical and systemic administration, and rapid absorption circumventing the first-pass effect, the absorption of therapeutic proteins is still ineffective, largely due to the immunological and physicochemical barriers of the lungs. Most studies using spray-drying for the nanoencapsulation of drugs focus on the delivery of conventional drugs, which are less susceptible to bioactivity loss, compared to proteins. Herein, the development of polymeric nanoparticles by spray-drying for the delivery of therapeutic proteins is reviewed with an emphasis on its advantages and challenges, and the techniques to evaluate their in vitro and in vivo performance. The protein stability within the carrier and the features of the carrier are properly addressed.

## 1. Introduction

The recent advances in the areas of biotechnology and bioengineering have resulted in the emergence of proteins for various therapeutic applications, such as erythropoiesis stimulation, hemophilia, Gaucher’s disease, glucose regulation, carcinomas, among others [1,2,3]. Most therapeutic proteins are administered through the parenteral route which is uncomfortable for patients, because of the use of needles and injectable materials, leading to poor patient compliance [4]. The oral route is generally preferred, but the poor oral bioavailability of therapeutic proteins hinders their oral administration [5].

To overcome these problems, the lungs have been suggested has an appropriate delivery route due to the lungs’ physiologic features [6]. The lung has a large surface area and a well vascularized thin epithelial lining, which provides a noninvasive method for therapeutic proteins delivery, a direct access to systemic circulation and a way to avoid first pass metabolism and degradation by gastrointestinal tract [7,8]. Despite the advantages, the pulmonary delivery of therapeutic proteins has also limitations because of their high molecular weight and difficulty to cross biological membranes, their poor bioavailability due to immune response, enzymatic degradation and lack of specific targeting [9].

A promising strategy to overcome these drawbacks is to encapsulate proteins into suitable delivery systems. In this context, nanomedicine in the field of pulmonary protein delivery has emerged as a technological approach that uses nanotechnology and pharmacotherapeutics for the delivery of therapeutic proteins [10]. Nanoparticles are solid colloidal particles constituted by macromolecular substances and range in size from 10 nm to 1000 nm. Based on their morphology, nanoparticles can be categorized in nanospheres that have a homogeneous and matrixial structure in which the drugs are uniformly dispersed, and nanocapsules that present a typical core-shell structure [11,12,13,14]. Nanoparticles are made of various materials such as natural or synthetic polymers, metals or lipids and they are recognized as useful drug carriers in general, showing good therapeutic properties [14].

Polymeric nanoparticles have been developed for pulmonary delivery, due to their biocompatibility, ability to enter intracellular compartments, their sustained drug release, enhanced drug stability and absorption, and targeted delivery [15]. Several techniques are being developed to produce nanoparticles with properties suitable to provide drug delivery to the lung [6]. Spray-drying is a well-established method commonly used in formulation development to produce dry powders from a liquid sample. Nanoparticles produced by spray-drying undergo gaseous hot drying to remove the water from the formulation, thus increasing long-term stability and shelf-life [16,17]. In addition, in the last years, the spray-drying has been demonstrated to be a suitable method for the preparation of proteins intended for pulmonary delivery [5,18].

The aim of this review is to perform a full overview on the spray-drying of protein-loaded polymeric nanoparticles for dry powder inhalation. Thus, it will be discussed the benefits of protein encapsulation into nanoparticles, and their formulation by spray-drying into an inhalable dry powder. The different processing features involved in this technique, and its consequences to the dry powder features will be focused. More importantly, different protein-loaded polymeric nanoparticles and their in vitro, ex vivo and in vivo performance, as well as the toxicity problems involved in their administration will be fully disclosed.

## 2. Anatomy and Physiology of the Respiratory System

**Figure 1:**
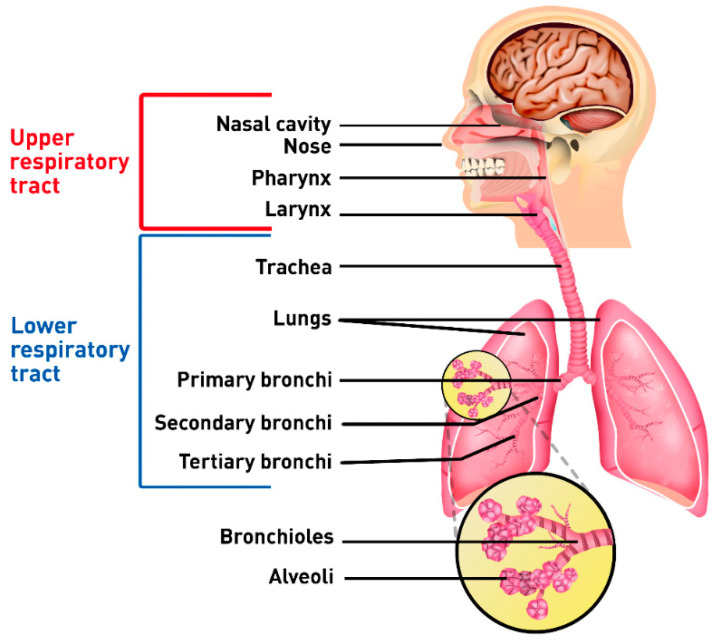
Anatomical representation of the human respiratory system.

The anatomical and physiological features of the respiratory system make it suitable for topical and systemic drug delivery [6]. The upper respiratory tract is formed by the nose, nasal cavity, pharynx, and larynx, which form the upper airways (Figure 1). The lower airways are composed by the trachea, lungs and bronchi [19]. The upper respiratory tract filters the particles that reach the lower airways providing protection [20].

The respiratory tract is lined by the bronchial epithelial varying from 3–5 mm in thickness, followed by the bronchiolar epithelium between 0.5–1 mm. The serous fluid and mucus coat the lumen of the bronchial airways to protect the epithelium from penetration of particles. The mucus is removed by the movement of cilia by a process called pulmonary clearance [6,21]. The thin epithelium allows the exchange of oxygen and carbon dioxide between blood and the inhaled air, the carbon dioxide exchanges allow the blood to maintain the pH at physiological levels [6,19]. The trachea is connected to the primary bronchi, which are further divided into secondary and tertiary bronchi and bronchioles. The bronchioles continue to branch into terminal bronchioles, which are the smallest airways without alveoli. The airways from the trachea through the terminal bronchioles are the conducting airways, directing the inhaled air to the distal gas-exchanging regions of the lungs, gradually heating, and humidifying the air as it flows through them. The branching from the terminal bronchioles culminates into the respiratory bronchioles. Lastly, the respiratory bronchioles branch into the alveoli. The respiratory zone includes the region from the respiratory bronchioles to the alveoli [22].

The alveoli are the functional unit of lungs and approximately 300 million alveoli are present in the lungs. Moreover, capillaries overlay the alveolus wall, enabling a large surface-area for gaseous exchange. During inspiration, oxygen diffuses from the alveoli into the blood, whereas during exhalation, the blood releases carbon dioxide to the alveoli. The alveolar epithelial cells type I and type II pneumocytes coat the alveolar wall. The type I cells are small, non-phagocytic, membranous pneumocytes and share a basement membrane with the pulmonary capillaries. The type II cells are large, granular, epithelial pneumocytes and secrete lung surfactant to prevent alveolar collapse. The alveoli have also macrophages, which clear large particles [6,19,22,23,24,25].

### 2.1. Particles Deposition and Clearance

**Figure 2:**
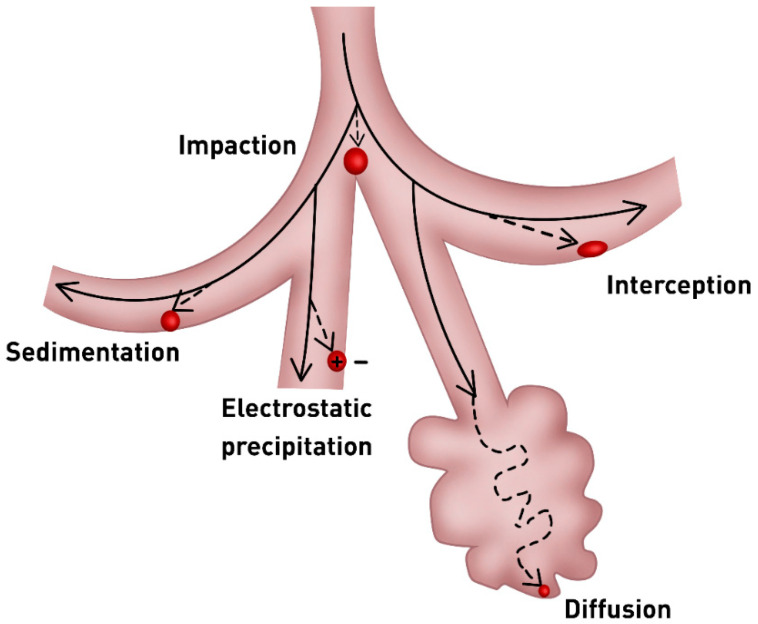
Particle deposition mechanisms into the respiratory tract. The solid line represents the air flow, and the dashed line represents the particles trajectory.

The inhalation of particles and their deposition within the several regions of the lungs is influenced by the particles physicochemical characteristics, such as size, morphology, charge, and hygroscopicity, and also by the breathing pattern, flow rate and tidal volume [21,26]. Particles reach the lungs through different mechanisms including impaction, sedimentation, and diffusion (Figure 2), however interception and electrostatic precipitation have also been described [27].

Particle size plays a key role in their deposition region; particles with a mass median aerodynamic diameter (MMAD) larger than 5 µm are deposited in the upper airways by a process called impaction. In this process, particles are led to the oropharynx by the airflow changes in the airways bifurcation and centrifugal forces [19,21,28]. Thus, for drug delivery into the whole lung it is required particles with a MMAD below 5 µm. For the alveolar epithelium delivery, particles with an MMAD lower than 3 µm are required [29]. Particles with an MMAD inferior to 0.5 µm are deposited in the alveoli by the Brownian motion in low air flow, although their low inertia may also cause them to be removed [21,23,30].

Particles that are elongated, such as fibers, have been suggested to be more efficiently deposited because they travel the airways following the breathing airflow and are deposited in the lungs by interception, that adhere to airway wall upon contact. The electrostatic precipitation is related to the particles charge. The charged particles that are close to airways surfaces induce image charges of opposite sign onto the surface and consequently, these particles are electrostatically attracted to the airway walls and deposited on them. The deposition through electrostatic precipitation is quite small, in comparison with the deposition by the preceding mechanisms [31,32,33,34].

Once deposited, particles are subjected to the existing clearance mechanisms in the respiratory system [35]. There are several clearance mechanisms that operate in different regions of the respiratory system to eliminate the inhaled particles [27,35]. The mechanical clearance is the first clearance mechanism and it takes place by sneezing, coughing or swallowing of the deposited material still within the nasal or oral pathway, followed by the mucociliary clearance, which is the primary clearance mechanism to remove insoluble deposited particles in the upper airways. The mucociliary escalator transports the particles-loaded mucus towards the larynx, where it can be swallowed to the gastrointestinal tract or removed through the mouth as sputum [21,27]. The cilia movement, and mucus composition highly affect the clearance of substances [31].

The insoluble particles that reach the lung alveolar region are subjected to the macrophage-mediated clearance. Thus, particles are phagocytosed by macrophages and transported through the alveolar surface and undergo translocation to the lymphatic system or degradation by the intracellular enzymatic lysosomal system [27,35]. The alveolar macrophages are less efficient in the phagocytosis of ultrafine particles compared to larger particles, as well in the phagocytosis of fibrous particles comparing to spherical particles. Likewise, small particles that form large aggregates are easily phagocytosed, whereas individual particles can escape from the macrophage clearance [36,37].

### 2.2. Barriers to Pulmonary Uptake of Proteins

Modern carriers seem promising to deliver large peptides and proteins due to the systems features, however, their absorption through the lungs is still poor leading to lack of therapeutic effect. The pulmonary uptake of proteins is affected by its physicochemical properties, such as the isoelectric point and formulation pH, ionic strength, solubility and concentration [38]. They may influence the structural stability and charge of proteins influencing its uptake.

The mucus, mucociliary clearance, alveolar lining layer and epithelium, basement membrane, pulmonary enzymes and macrophages as the main barriers to pulmonary uptake of therapeutic proteins and peptides [39]. The alveolar epithelium and capillary epithelium are permeable to small hydrophilic molecules (ranging from 100 to 1000 Da and log P < 0), gases, and lipophilic compounds (log P > 0). The hydrophilic drugs are assumed to permeate the aqueous pores in the intercellular tight junctions, whereas lipophilic drugs permeate the lungs via the transcellular pathway. Large molecular size drugs to and ionized molecules have limited permeation [40]. Once in the lung, the macrophages secrete proteases, inflammatory, and immunomodulatory mediators. Proteins that reach the alveoli are usually degraded and removed by alveolar macrophages. Thus, macrophages hinder the transport of large proteins, avoiding their uptake to the bloodstream. The mucus that lines the pulmonary epithelium and the surfactant lining the alveoli are rich in protease inhibitors and pose a physical a barrier to the diffusion of peptides and proteins. It has been suggested that they also contribute to the slow degradation of proteins, however, this finding is controversial as membrane-associated (epithelial and endothelial), intracellular proteases and peptidases readily degrade delivered proteins [4,41].

## 3. Advantages and Disadvantages of Protein Loading into Nanoparticles

Nanoparticulate systems possess interesting features to circumvent the shortcomings of lung drug delivery, such as improved bioavailability, biocompatibility, minimal side effects, decreased toxicity to other organs, tissues and cells, sustained and targeted delivery, and reduced cost [42]. Moreover, nanoparticles can be used to encapsulate a variety of therapeutic agents and release the active molecules in a controlled manner [11].

The transport of therapeutic proteins into the body is limited by: (i) their high molecular weight, which prevents the crossing of tissue barriers; (ii) their short lifetime due to immune response, enzymatic degradation; and (iii) lack of capability to deliver to a target area. These barriers can be overcome by loading the therapeutic proteins into nanoparticles [9,43]. The encapsulation of proteins into nanoparticles presents great advantages, including the protection of the protein from degradation and maintained bioactivity, sustained release and reduced toxicity. Therapeutic proteins have a short half-life because of proteolysis and rapid clearance from the body, leading to repeated administration. Therefore, nanoparticles protect encapsulated proteins from the action of proteases by increasing their residence time in bloodstream, and hence their bioavailability, thus decreasing the number of necessary administrations. The encapsulated proteins also interact with the nanoparticles material by forming a coat or adsorbing onto their surface, or by bioconjugation, allowing that therapeutic proteins cross biological barriers, such as the pulmonary epithelium. Furthermore, nanoparticles promote the targeted delivery of proteins to specific tissues or cells [9,43,44,45].

Despite the several advantages of the encapsulation of proteins into nanoparticles, during the encapsulation and delivery processes, proteins may undergo changes in structure conformation and loss of activity, hindering their bioactivity and consequently having reduced therapeutic effect [9,43,44]. The advantages and disadvantages of nanoparticles as carriers for therapeutic proteins delivery are summarized in Table 1.

## 4. The Spray-Drying Technique

Nanoparticles can be produce by three main methods: (i) physicochemical methods, which consist in induce the precipitation of preformed polymers by emulsification; (ii) in situ chemical synthesis methods of macromolecules, such as polymerization or interfacial polycondensation reactions; and (iii) mechanical methods, such as spray-drying [13].

**Figure 3:**
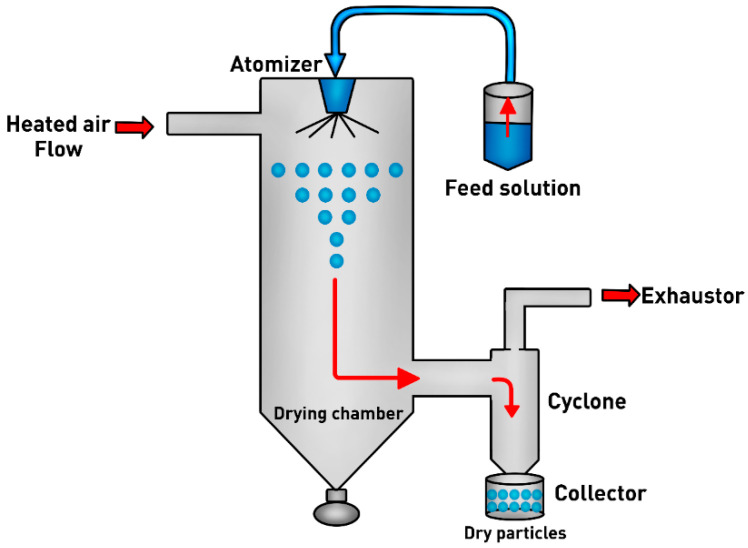
Scheme of the spray-drying process for dry particles production. Adapted with permission from [46], Elsevier, 2015.

Spray-drying is a process based on the conversion of a liquid material into a dry powder, by atomizing a solution, emulsion or suspension into a hot drying gas medium that is commonly air [16,46]. The spray-drying process is represented in Figure 3 and consists in four fundamental steps: (i) atomization of liquid feed into a spray; (ii) spray droplets mixing by a heated gas stream; (iii) formation of dry particles by evaporation of the liquid; and (iv) dry particles collection and size separation [16,30,46,47].

A liquid feedstock is fed into the drying chamber by a peristaltic pump through an atomizer or nozzle initiating the atomization. This step generates spray (small droplets with micrometer scale), which are subjected to the sufficient temperature for fast solvent evaporation to take place, leading to the formation of dry particles. The dry particles are separated from the drying gas by means of a cyclone that deposits them in a collector situated at the bottom of the device [5,46].

Sprays are produced either by particle atomization with pressure, centrifugal, kinetic, and piezoelectric energy. The energies used are dependent on the atomizer type; a pressure nozzle atomizer produces particles by pressure, a rotary atomizer produces particles by centrifugation, a two-fluid nozzle atomizer produces particles by kinetic energy between the two fluids, and a piezoelectric atomizer produces particles using piezoelectric energy [5,30,48]. In addition, a special apparatus, the Nano Spray Dryer B-90, provides a nanotechnology to obtain protein nanoparticles for drug delivery applications [5]. The spray-dryers can operate in a co-current of cold air, and it is used for drying heat-sensitive materials, counter-current, where the final product is in contact with the hottest air, and it cannot be used with temperature-sensitive materials or mixed flow manner. Besides that, it can also operate in different modes such as open-cycle (uses air as drying gas that is not re-circulated), closed-cycle (uses an inert gas that is re-cycled in the drying chamber) and semi-closed cycle, with or without aseptic control. The spray-drying process must be adjusted to obtain dry particles with desired properties [17,30,46]. For example, a high flow rate of the liquid feed, large nozzle diameter and high formulation concentration leads to the formation of larger particles, while a low surface tension, high atomization pressure and small nozzle diameter favor the formation of smaller particles [46,49,50].

### 4.1. Advantages and Drawbacks of Spray-Drying

Spray-drying is a very appealing technique used to produce particles, with many advantages over common techniques, such as emulsion/solvent evaporation, nanoprecipitation and freeze-drying. Spray-drying is a relatively simple, fast, continuous, reproducible, scalable and cost-effective process [46,51]. It is a single-step, drying method proper for heat-sensitive drugs such as therapeutic proteins, that allows to control particle size and morphology. The rapid solvent evaporation has a cooling effect on the formulation, despite the high temperatures during the drying process, because evaporation is an endothermic reaction [52]. This technique is suitable for heat-sensitive compounds because small droplets are formed during the atomization, leading to fast solvent evaporation due to the high surface-area. The droplets are exposed to high temperature for a very short time during the drying process [46].

The spray-drying is a dehydration process commonly used to prolong the lifespan and bioavailability of the product. Similarly to freeze-drying, the water removal from formulations contributes to long-term stability [53,54,55]. This drying process is attractive to produce drug-loaded polymeric nanoparticles, contributing to the high efficacy of the final particulate dosage form. Furthermore, spray-drying has been employed in the production of inhalable dry powders for pulmonary drug delivery, such as therapeutic proteins. The particle size and morphology can easily be controlled by spray-drying by controlling the process factors and formulation composition [16,46,52,56]. 

Despite the several advantages of the spray-drying technique, it also presents a variety of drawbacks, since spray-drying requires hot dry air that might cause thermal stress to some proteins, thus contributing to their instability and loss of their native structure. Additionally, the dehydration process involved in this technique may cause structural modification and protein denaturation due to shear stress (e.g., by nozzle atomization), which may affect particle stability. This may be overcome by the loading of therapeutic proteins into nanoparticles. Furthermore, the production yield strongly depends on the work scale, thus in lower scale setups (e.g. laboratory scale) the yield of production is typically low, because of loss of product in the walls of the drying chamber. Large sample volumes are also required, and consequently require higher evaporation rates of liquid. Furthermore, high pumping power is needed to pump the liquid feed [46,52,56]. The advantages and drawbacks of the spray-drying technique are summarized in Table 2.

### 4.2. Requirements for Inhalable Dry Particles

Particles for inhalation should meet some specific requirements: neutral pH, isotonicity, biocompatibility, good aerosolization characteristics such as, appropriate size for lung delivery through the air flow and scaling-up potential [33,57]. To calculate the efficiency of pulmonary drug delivery, the emitted dose, dose delivered to the lung and bioavailability must be determined. Emitted dose and dose delivered to the lung are commonly determined in vitro using a multistage cascade impactor, and it is predominantly ruled by properties of the particles and delivery device. The particle size and morphology affect the dissolution rate and, consequently the in vivo permeability of the drug, drug metabolism, and its bioavailability. Furthermore, phagocytic clearance in the lungs decreases the bioavailability of the drug [30].

The success of the therapeutic protein delivery lies with dose uniformity and high emitted doses being delivered. Thus, particles should have high physical and chemical stability, appropriate MMAD, low surface energy and charge, a relatively narrow particle size distribution, and should be readily aerosolize at relatively low aerodynamic dispersion forces. The particles with low density are advantageous for pulmonary delivery due to their large volume diameter and small aerodynamic diameter. The particle low density combined with small aerodynamic diameter leads to increased dispersibility and in depth lung delivery [30]. Moreover, large porous particles can escape the natural phagocytic clearance compared to solid particles of similar size [30,58]. For higher therapeutic efficacy, rapid dissolution, absorption, biological molecules stabilization, amorphous crystalline state of the particles are preferably used [30]. Particle aggregation is an undesirable occurrence caused by electrostatic, van der Waals and capillary interactions between particles that affect the formulation stability and efficacy. These interactions can be avoided using excipients with preferential absorption onto the particle surface, such as dipalmitoyl phosphatidylcholine (DPPC) [30,59,60].

## 5. Production of Inhalable Polymeric Nanoparticles

Nanocarriers for drug delivery by inhalation have been described in the literature featuring different matrices, such as lipid or polymer-based micelles, dendrimers, liposomes and polymeric nanoparticles [26]. Among these nanocarriers, polymeric nanoparticles have been preferred to incorporate drugs due to their ability to bear high drug loading and modify their pharmacokinetics [61]. The properties of most polymers can be modified (i.e., polymers can be surface-modified, co-polymerized or bioconjugated), which makes polymeric nanoparticles a versatile drug delivery system that can be tailored to penetrate biological barriers, delivering drugs to tissues or even into intracellular compartments. Furthermore, the polymers protect the loaded drug against degradation while providing control over the release kinetics, without affecting the normal cell function [9,10,26,61]. The drugs can be adsorbed on the surface of the polymer, encapsulated in polymer-based carriers, or dispersed in a polymeric matrix. The polymeric matrix slowly dissolves and releases the drug upon contact with the mucus and humid lung environment [26].

The polymeric nanoparticles should be made of polymers that are biocompatible, cause no immunogenicity and have good clearance from the body [43]. Commonly used polymers for pulmonary drug delivery include animal or algae derivatives (e.g., gelatin, alginate, chitosan, albumin) and synthetic polymers (e.g., poly lactic-co-glycolic acid (PLGA), polyethylene glycol (PEG), and poloxamers) [9,10,62].

Gelatin is a collagen derivative widely used to produce nanoparticles due to its high loading efficiency of drugs. The loading efficiency is due to the particle surface having carboxyl and amino groups that bind to the available groups of drug molecules [10,43,63]. Alginate, extracted from brown algae, is a hydrophilic polymer that forms arranged polymeric gel networks with divalent ions at room temperature. Its hydrophilic nature and biocompatibility enable the incorporation of hydrophilic drugs and the negative charges on the surface allows higher encapsulation efficiency of positively charged molecules and coating. Furthermore, diffusion rates of drugs through the hydrogel matrix can be easily controlled and attain sustained drug delivery of several molecules [64]. Chitosan is a linear polysaccharide with mucoadhesive properties with permeation enhancement properties of large molecules across mucosal surfaces, facilitating nanoparticle retention in the lung after administration [43,62,65].

Albumin is a globular protein which can be obtained from different sources including bovine and human serum albumin and ovalbumin from egg white. This protein is water-soluble, involved in the transport of nutrients through molecule binding. Albumin is also soluble in salt solutions and relatively stable when heated up to 60 °C up to 10 h, without showing denaturation effects [43,66]. Moreover, this globular protein carries reactive groups (e.g., thiol, amines, and carboxyl), which can act as ligand binding unit by a covalent linkage or surface modifier. The therapeutic drug entrapped in the albumin nanoparticle can thus be easily digested by the enzyme protease [66].

PLGA is a biocompatible polymer with improved colloidal stability and controlled release properties, allowing sustained drug release over a period of weeks to months depending on the ratio of monomers used and minimizing fluctuation of drug concentration, without causing damage to the lung tissue [10,67]. PLGA can be easily modified with other polymers to develop complex copolymers (e.g., PEG-PLGA-PEG), which makes it one of the most useful polymers for design and development of controlled and targeted therapeutic drug delivery systems [9,68,69,70].

PEG is a non-ionic hydrophilic polyether and synthesized by polymerization of the monomer ethylene glycol, and improves the hydrophilicity, aerodynamic characteristics and retention time of nanoparticles. PEG is also a non-biodegradable polymer and undergoes unchanged renal clearance, however, it is highly biocompatible and does not accumulate in tissue [10]. Other synthetic polymers have been used to prepare drug-loaded polymeric nanoparticles, such as polylactides, polyglycolides, poly-*n*-butyl cyanoacrylate, poly ε-caprolactone, polyhydroxy butyrate, poly 2-hydroxyethyl methacrylate, poly-*N*-2-hydroxypropyl methacrylamide, polymethyl methacrylate, polyisobutyl cyanoacrylate, polyisohexyl cyanoacrylate and polyvinyl alcohol [9,43].

### 5.1. Methods to Obtain Inhalable Nanoparticles by Spray-Drying

The polymeric nanoparticles for inhalation may be obtained by different ways. Overall, the nanoparticle suspensions may be previously prepared and further dried by spray-drying, or directly prepared during spray-drying upon passage through the atomizer and drying. It is important to notice that the small size of polymeric nanoparticles is not acceptable for direct administration into the lungs, since they may get retained in the upper airways or exhaled without reaching the deep lungs. Therefore, polymeric nanoparticles to be formulated into an inhalable dry powder need to be prior mixed with bulking agents or even encapsulated into microparticles, to attain an acceptable size for pulmonary delivery. In this section the different methods to obtain polymeric nanoparticles by spray-drying for dry powder inhalation are disclosed.

#### 5.1.1. Nanoparticle Formulations Dried by Spray-Drying

There are several methods to produce polymeric nanoparticles such as emulsification, nanoprecipitation, spray-drying, spray-freeze-drying, high-pressure homogenization, supercritical fluid extraction and others. Thus, polymeric nanoparticles may be previously prepared in a suspension form, and further spray-dried to improve its physicochemical stability and efficacy in a long term [26,52,61,71]. Different adjuvants may be added to the nanoparticle suspension to obtain easily re-dispersible powders with a good recovery of particle size in the nanoscale [72]. Guterres et al. prepared microparticles encapsulating polymeric nanoparticles by spray-drying to improve stability of the loaded drug [71]. Silicon dioxide was added to the formulation as a drying agent and as scaffold for the nanoparticles [72,73].

All the variables involved in spray-drying process, such as the solvent, solute concentration, physicochemical properties of the drug, inlet and outlet temperature, feed properties, pump rate, atomizing pressure, gas type, and gas flow rate must be carefully considered when designing the study to obtain polymeric nanoparticles with the desired properties to reach the target in the lung [26]. The use of different materials also leads to well defined complex architectures of particles and determines the pulmonary pathway of drug administration [71]. Still, the application of temperature to produce nanoparticles by spray-drying is the main problem, since it can lead to drug instability, particle aggregation and possible interference with drug entrapment into nanoparticles [74].

The spray-drying process may influence and change the particle size, so when converting a nanosuspension into inhalable and redispersible nanoparticles it is important to evaluate the ratio of nanoparticle sizes after and before spray drying, to evaluate possible particle size changes upon spray-drying [75].

#### 5.1.2. Spray-Drying of Solutions to Obtain Nanoparticles

The production of nanoparticles by spray-drying is a multi-step process that requires the production of a clear homogeneous solution with one or more constituents, before spray-drying [72]. The use of an organic solution in spray-drying allows to achieve lower water content in the nanoparticle dry powder [26]. Selvaraj and Messing prepared nanoparticles by spray-drying by combining a solution of a drug and adjuvants. They showed the effect of the operational conditions, drug physicochemical properties, adjuvants, and solvents on the nanoparticle size and distribution [76]. PLGA nanospheres have been successfully prepared by spray-drying to encapsulated estradiol metabolites and analogs using dichloromethane as a solvent [77]. Other methods include the use of chemical cross-linking agents to produce nanospheres. Marx and Gorodetsky prepared albumin nanospheres chemically cross-linked with glutaraldehyde by spray-drying. To reduce the formulation cytotoxicity, Factor VIII was included as the cross-linker and the nanospheres became suited to deliver proteins, peptides, antigens, and drugs [78].

Recent developments in the spray-drying field have introduced a novel technique to prepare nanoparticles called nano spray-drying [5,52,72]. In the nano spray-drying, a membrane is attached to the spray head to generate nanoparticles with homogeneous size by piezoelectric effect. The particles are then collected and separated by an electrostatic particle collector. This technique allows the use of small sample volume (2 mL), particle sizes approximately of 300 nm, and manufacturing yield of 90% [5,52].

#### 5.1.3. Spray-Drying of Dispersions and Emulsions to Obtain Nanoparticles

Despite the spray-drying technique is commonly used to obtain polymeric nanoparticles from colloidal aqueous suspensions or solutions, this technique has also been employed to prepare fine redispersible drug particles using dispersions or emulsions combined with polymers to stabilize the particles. The release rate of drugs from nanoparticles can be controlled by refining the ratios between hydrophilic polymers. Polymer concentration below 0.5% *w/w* increases the release of poorly water-soluble drugs in solid dispersions [72,79].

Overall, there are two different methods to obtain drug-loaded polymeric nanoparticles by spray-drying. A single-phase method, which consists in the use of carriers (e.g., cellulose derivatives or other hydrophilic polymers) as the matrix of nanoparticles containing a water-insoluble agent. This method used a solution where the drug was dispersed in the aqueous phase before spray-drying. The emulsion method includes two immiscible phases, where the drug was dissolved in the water-immiscible solvent and the carrier is solubilized in water. In a study comparing both methods, the single-phase method provided particle size of approximately 200 nm after spray-drying whereas the emulsion method achieved 5000 nm, in which the final particle sizes were directly proportional to the primary emulsion droplet sizes [80]. The particle size is related to the shearing method and rate, and time of shearing. Thus, higher shearing and longer time of emulsification generated smaller droplet sizes and, hence, smaller particle sizes.

Emulsions are defined as heterogeneous systems of two liquid phases where one of the liquids is the dispersant and the other is dispersed in the form of small droplets into the other. In general, to prepare a stable emulsion, an emulsifying agent, such as polymers (e.g., hydroxypropylmethylcellulose) are needed. The dispersed liquid is known as the internal, dispersed or discontinuous phase, while the other liquid is the external, dispersant or continuous phase [79,81,82]. Single or double emulsion methods can be used, depending on the hydrophilicity of the drug to be encapsulated. Hydrophobic drugs may be encapsulated by the single-emulsion method by dissolving simultaneously the drug and the polymer in a water-immiscible organic solvent to form the inner phase, and the solution is further emulsified in water and a surfactant as external phase. The hydrophilic drugs may be encapsulated in nanoparticles produced by double emulsion. In this type of emulsion, the drug is dissolved in the inner aqueous phase, and the polymer matrix is located in the middle phase, whereas the external phase is usually a surfactant solution [83].

## 6. Effect of Spray-Drying on the Stability of the Loaded Protein and Nanocarrier

As aforementioned, spray-drying may change the conformation of proteins that denature and suffer irreversible aggregation. Therefore, the biggest challenge of spray-drying is to find a suitable formulation composition and process conditions that avoid negative impacts of the multiple technique steps on protein-nanocarrier stability. In this process heat-sensitive materials are affected by several stresses compromising their stability. However, thermal denaturation of proteins during the drying process not only depends on the temperature, but also on the length of period of exposure to the hot drying air. Usually, the droplets are exposed to high temperatures for a few seconds and thermal denaturation is often considered negligible. The stability of protein-nanocarrier can also be influenced by the adsorption of proteins to various interfaces. This can result in the unfolding of their structure causing aggregates at the air–liquid interface. Moreover, water removal by dehydration processes might lead to structural modification and protein denaturation [52,84,85]. Structural changes of the loaded protein can be assessed by evaluating the integrity of its secondary and tertiary structure by Fourier transform infrared spectroscopy, far-UV circular dichroism, fluorescence spectroscopy and Raman spectroscopy [55,86,87].

To avoid instability of protein-nanocarriers dried by spray-drying various strategies have been applied, including process optimization using multivariate analysis and addition of disaccharides or surfactants in the liquid feed. Several sugars, like mannitol, lactose, sucrose, trehalose, raffinose, and surfactants such as Tween 80 have been widely used to preserve the stability of proteins during the spray-drying process. In addition, a careful selection of process parameters, such as temperature and atomization rate, allow the preparation of protein-loaded nanocarriers with minimal protein damage, suitable residual moisture content, good aerodynamic properties, and satisfactory storage stability [30,52]. Lee et al. have correlated the effects of spray-drying process and formulation parameters on the characteristics of the final particles [5].

## 7. Performance of Inhalable Protein-Loaded Polymeric Nanoparticles

In this section different studies on protein-loaded polymeric nanoparticles delivery are discussed. Also, the in vitro, ex vivo and in vivo models to evaluate their performance will be focused. Several proteins and peptides, such as insulin, calcitonin, alpha 1-antitrypsin and exendin-4 have been successfully loaded in polymeric nanoparticles and used for pulmonary delivery. The physicochemical characteristics of inhalable protein-loaded polymeric nanoparticles described in this section are depicted in Table 3.

Makhlof et al. have evaluated the role of the calcitonin-loaded glycol chitosan nanoparticles and thiolate derivative of glycol chitosan, in enhancing the pulmonary absorption of the incorporated peptide. It was shown that both nanoparticles of glycol chitosan and its thiolate derivative significantly improved the pulmonary absorption of calcitonin, with a pronounced hypocalcemia effect for a period of 24 h and 12 h and enhanced pharmacological availability of 27% and 40%, respectively. The increase in protein absorption was attributed to the mucoadhesive and permeation enhancing effects of glycol chitosan [88]. In another study, Pirooznia et al studied the performance of alpha 1-antitrypsin-loaded PLGA nanoparticles and verified that PLGA nanoparticles had a high efficiency entrapment of about 90% and released 60% of alpha 1-antitrypsin in 8 h [89]. Thus, alpha 1-antitrypsin-loaded PLGA nanoparticles may be considered a promising formulation for the treatment of many respiratory diseases. Al-Qadi et al. prepared chitosan nanoparticles for systemic delivery of insulin via inhalation [15]. Mannitol was added to the formulation as an adjuvant to improve nanoparticles size distribution and achieve deposition in the deep lungs. The feasibility for systemic delivery was tested in rats after intratracheal administration, so glucose levels were collected, showing prolonged hypoglycemic effect and proving the system suitability for systemic pulmonary delivery of therapeutic proteins.

Zhao et al. have also prepared polymeric nanoparticles with gelatin, d,l-glyceraldehyde and poloxamer 188 for the pulmonary administration of insulin [90]. A novel water-in-water technique was developed to produce the gelatin nanoparticles. 

Nanoparticles displayed a particle size of about 250 nm and zeta potential of −21.2 mV at a gelatin-poloxamer ratio of 1:1, and were administered to mice by intratracheal instillation. The alveolar deposition of the formulation was confirmed by microscopy and a fast-hypoglycemic effect was observed in vivo. A sustained drop in glucose was observed in the group treated with the formulation up to 6 h, contrasting with the control group.

PLGA nanoparticles coated with chitosan were described by Lee et al. for the delivery of palmitic acid-modified exendin-4 (Pal-Ex4). Adenocarcinoma human alveolar epithelial cells (A549) were injected in a mouse model and the PLGA nanoparticles were tested. Non-coated PLGA nanoparticles showed lower intake by cells compared to the chitosan coated PLGA nanoparticles. Similarly, the coating with chitosan delayed the release of Pal-Ex4 1.5 days compared to the non-coated nanoparticles. Chitosan coated PLGA nanoparticles were present in the lungs 72 h after administration, resulting in a 3 fold higher hypoglycemic effect, showing the potential of this formulation to treat type II diabetes [91].

### 7.1. In Vitro Simulated Lung Fluids

Simulated lung fluids (SLF) are solution models to evaluate the interaction between drugs and the human lung fluid to determine in vitro and in vivo correlations [92,95]. SLS composition is varied and several solution compositions have been proposed as acceptable models, including phosphate-buffered saline (PBS, pH 7.4), Gamble’s solution (pH 7.4), modified Gamble’s solution, and artificial lysosomal fluid (ALF, pH 4.5). The solutions at pH 7.4 mimic the extracellular environment in ionic strength and pH, and therefore, are used to test the interaction of particles with the lung fluid and surfactants. The ALF at pH 4.5 simulates the conditions in the extracellular matrix and the intercellular pH of the lung cells [96,97,98].

In a previous work, Menon and co-workers screened six polymeric nanoparticles: gelatin, chitosan, alginate, PLGA, PLGA-chitosan, and PLGA-PEG, for delivery of bovine serum albumin (BSA) as a protein model and assessed the stability of nanoparticles in Gamble’s solution [10]. Gelatin, chitosan, alginate, PLGA, PLGA-chitosan displayed a size of approximately 300 nm and a two-stage release profile. The PLGA and gelatin nanoparticles remained stable, i.e., maintained consistent particle sizes without aggregation, in deionized water, serum, saline and SLF (Gamble’s solution), up to 5 days, thus indicating high stability. On the other hand, chitosan and alginate nanoparticles showed fluctuations in size, indicating nanoparticle aggregation or polymer degradation. The authors described that chitosan becomes unstable at pH 7 due to thermodynamic instability of the system. Therefore, this study suggested that chitosan and alginate nanoparticles are less favorable for pulmonary delivery as their instability may lead to inflammation and reduced efficacy of the therapeutic proteins due to faster clearance from the lung. These results agree with previous studies that assessed the stability of polymeric nanoparticles [99,100,101,102,103].

A recent study performed by Ghasemi and co-workers compared the effect of using Gamble’s solution or ALF in the stability of chitosan-genipin nanohydrogel incorporating alpha-1 antitrypsin [92]. Results showed lower α-1 antitrypsin release from nanoparticles exposed to Gamble’s solution at pH 7.4 compared to ALF at pH 4.5 which is probably due to the reduced electrostatic interactions between the protein and nanoparticles at pH 4.5. Nevertheless, Hanks balanced salt solution (HBSS) and Dulbecco’s modified Eagle’s medium have also been used as models for the lung fluid [104].

### 7.2. In Vitro Lung Epithelial Cell Culture Models

Cell culture studies are crucial to test preliminary outcomes prior to testing formulations in animals. Besides their availability and low cost, cell culture studies are relatively simple and allow to evaluate the performance of multiple experiments, reducing the number of animals needed for the in vivo studies [28,105].

Cell lines, which are less differentiated than primary cells, are often used for the assessment of general cellular effects and permeation of nanoparticulate drugs [32]. Several pulmonary epithelial cell lines have been studied to develop a standard cell line with the purpose to study the transport mechanisms across the pulmonary epithelium derived from both human and murine tissues, establishing it as lung-equivalents to Caco-2 [28,32,105,106]. Lung epithelial cell lines from human sources, such as A549, Calu-3, and 16HBE14o- are described in Table 4.

The A549 cell line is widely used as a model of the alveolar type II pulmonary epithelial cells where the cytochrome P450 is involved in the metabolization of xenobiotics. For this reason, the A459 is commonly used in metabolomics studies and in studies to assess the drug transport mechanisms [107]. Calu-3, 16HBE14o-, and BEAS-2B are the most used lung epithelial cell lines for the assessment of the bronchial barrier. These three cell lines are frequently used for drug absorption studies, to assess nanoparticle-cell interactions and to investigate the toxicity of nanoparticles [32,108]. Calu-3, a human sub-bronchial gland cell line, was also characterized by Foster to study drug permeation and transport mechanisms of drug molecules by pulmonary route [109]. Forbes et al. established the human bronchial epithelium cell line 16HBE14o- model to study the permeation mechanism and drug diffusion within the lungs. They investigated the effect of cell seeding density, collagen substratum and time on the development of barrier properties and permeability across the 16HBE14o- cell layers. The results showed that small hydrophilic molecules (Mw < 250 Da, log P < 1.9) had increased permeation in the 16HBE14o- model compared to the alveolar epithelium cell model. Furthermore, a sigmoid dependence was found between permeability and lipophilicity, suggesting that 16HBE14o- has selective permeability properties dependent on the solute physicochemical properties [106].

Kuehn et al. developed a new method to immortalize human alveolar cell lines via viral transduction of immortalized genes. The new cell line, human alveolar epithelial lentivirus immortalized (hAELVi), replicate the air-blood superficial tension and are used to study the absorption and toxicity of drugs and nanoparticles [110]. Other types of cell lines to model in vitro lungs include rat cells lines (e.g., SOPC1) and rabbit cell lines [28,111]. Often, lack of in vitro to in vivo correlations are observed, so animal studies must be carried out to confirm the in vitro findings [108].

### 7.3. Ex Vivo Lung Tissue Models

They are used prior to in vivo studies to assess the drug absorption mechanisms and distribution kinetics. Such models enable the development of simulation and predictive equations to perform in vitro/in vivo correlations. The ex vivo tissues commonly used are precision cut lung slices (PCLS) and isolated perfused lung (IPL) [28,105].

The IPL lung tissue is harvested from rats, rabbits and guinea pigs, and is kept in physiological artificial conditions in Krebs-Ringer or Krebs-Henseleit buffer solutions, at 37 °C, under 12–15 mL/min perfusion flow, in which oxygen and carbon dioxide are dissolved to mimic in vivo conditions [28]. This ex vivo lung tissue model has some advantages over other models such as the elimination of the first-pass effect, retention of the most lung tissue physiological properties and better control of experimental parameters; therefore, IPL is a much closer representation of the in vivo state than in vitro lung cell model. Nevertheless, the ex vivo lung tissue model can only maintain its viability for 3 h at 37 °C under physiological conditions, and for this reason its use in research is limited. This model requires great skill and precision during the surgery for removing the intact lungs from the animal. IPL models have been used to assess the permeation of drugs and the translocation of nanoparticles through the air-blood barrier [28,32,105,108].

Beck-Broichsitter et al studied the uptake of inhalable 5(6)-carboxyfluorescein (CF)-loaded polymeric nanoparticles in an isolated rabbit lung model. The nanoparticles had a size of 195 nm, narrow polydispersity index (PdI) of 0.225, a zeta potential (ZP) of −28.3 mV, and association efficiencies up to 60%. After deposition of the CF-nanoparticles in the IPL, a lower concentration of CF was detected in the perfusate (9.2 ± 2.4 ng/mL) compared to the CF solution (17.7 ± 0.8 ng/mL). The results suggested that the delivery of inhalable drug-loaded polymeric nanoparticles was a viable and a better approach for lung delivery [112].

The PCLS models are obtained from murine animals and by fixing and slicing tissue in frozen agarose solution using a tissue slicer, and washing the slices in cell culture medium to retain cell viability [28,113]. The cell viability is kept for 3 days which allows to study the endocytic mechanisms and inflammatory response. Specific areas of interest can easily be isolated, such as blood vessels and the alveoli, for mechanistic and physiology studies, including vasoactivation assessment caused by drugs or nanoparticles [28,113,114].

### 7.4. In Vivo Models

**Figure 4:**
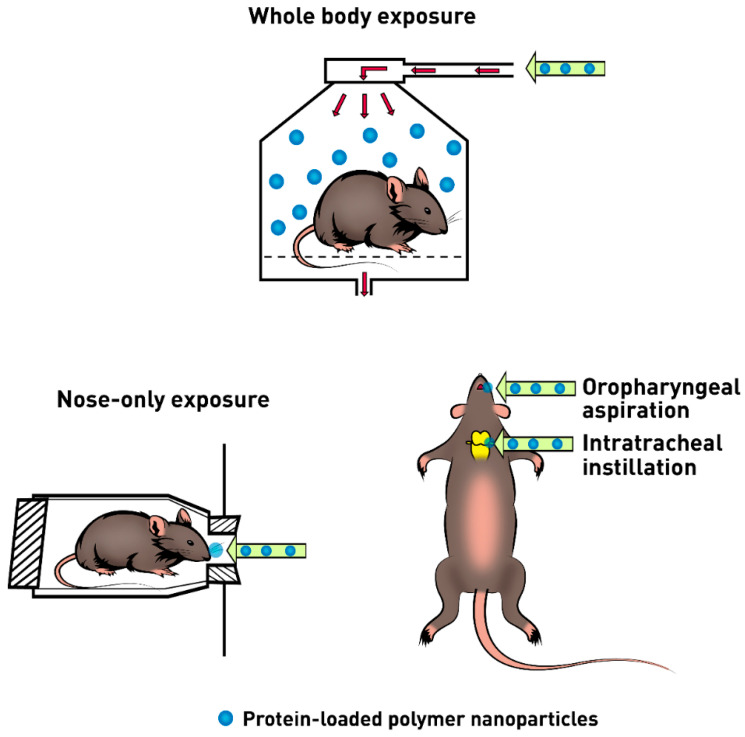
In vivo methods for protein-loaded nanoparticles pulmonary administration in small rodents.

The use of large animals is more desirable when carrying out in vivo experiments of inhalable systems for the simplicity of administration, easy access, and sample collection. Rabbits, pigs, dogs, monkeys, and sheep are commonly used in pharmacokinetics and efficiency studies, although small rodents have been described in the literature as acceptable animals for in vivo experiments [28,105]. The administration of formulations can include the whole body, head/nose/mouth-only or lung-only exposures (Figure 4) [108].

The whole-body exposure (Figure 4) is the method that causes less animal suffering, because does not require anesthesia nor surgery, being the most suited method for chronic exposure studies. It also mimics environmental, occupational, or intended exposure more realistically. The quality of the obtained results depends on the equal distribution and a steady concentration of the particles in the exposure chamber over time. The chamber must be saturated with the material being tested which requires large amounts of sample to be used, making the process costly. Furthermore, the animals can avoid exposure by huddling together in corners of cages or in the fur of other animals, affecting the results [32,108].

The head/nose/mouth-only exposure (Figure 4) causes stress to animals due to their food and water supplies removal during the exposure. Still, it is an efficient administration technique for formulations that require control and precision of doses to be administered. Furthermore, this method does not require anesthesia and surgery [108].

Lung-only exposure is the most used in vivo method and it is achieved by intratracheal instillation (Figure 4) requiring tracheotomy. Intratracheal instillation consists in the administration of 10–200 µL of a test solution through an incision made between the tracheal rings. Alternatively, nanoparticles may also be delivered by direct injection or oropharyngeal intubation through spray of the dosing solutions [32,105,108]. Although less effective, the oropharyngeal aspiration (Figure 4) is also used to achieve lung-only exposure, however, only a small dose or volume of the formulation can be administered, leading to low administration doses to be available for absorption and distribution. It has been suggested to be effective in the distribution of polystyrene nanoparticles in the lungs [32,115]. The lung-only exposure is an invasive method in which the formulation is directly deposited in the lungs and can cause tissue injury and heterogenous distribution of the drug within the lungs. Furthermore, lack of pharmacodynamic effect is observed in the system and autonomous nervous system due to anesthesia. This technique is often used for the delivery of protein-loaded nanoparticles and in the assessment of their toxicity, however, its limitations restrict their use as lung-only exposure poorly describes occupational and environmental exposure [108].

Kawashima et al. studied the in vivo effect of nebulized PLGA nanospheres loaded with insulin administered through intratracheal instillation to fasted guinea pig. Upon delivery of insulin-loaded PLGA nanospheres to the lungs, the systemic levels of glucose showed a significant reduction, leading to hypoglycemia over 48 h, in comparison with the aqueous solution of insulin, which only showed a basal glucose level during 6 h after administration, being rapidly recovered to the initial level [93]. Zhang et al. administered by intratracheal instillation polybutylcyanoacrylate nanoparticles loading insulin to rats at various doses. The lowest dose of used of 5 IU kg-1 produced a drop in blood glycaemia of 46.9% and prolonged the hypoglycemic effect [94]. In another study Menon et al. used gelatin and PLGA nanoparticles to test the behavior of these two formulations for pulmonary protein/deoxyribonucleic acid (DNA) uptake in vivo, following inhalation in rats [10]. Plasmid DNA encoding yellow fluorescent protein (YFP) and rhodamine-conjugated erythropoietin were selected to be incorporated into polymeric nanoparticles. It was observed and increasing pulmonary YFP expression that persisted for up to 7 days following nebulization, while erythropoietin presented a widespread pulmonary distribution that was observed for up to 10 days post-inhalation for both polymeric nanoparticles. In addition, the tissue expression for both complementary DNA (cDNA) and protein was uniform following nebulization of PLGA and gelatin nanoparticles. Nevertheless, YFP was highly expressed in the presence of PLGA than gelatin nanoparticles, along with a more sustained erythropoietin fluorescence using PLGA instead of gelatin nanoparticles. These findings suggested that both tested polymeric nanoparticles have potential for inhalable delivery of proteins and DNA, however PLGA nanoparticles are more effectively retained in the distal lung under physiological conditions.

Despite the established role of in vivo experimentation and advantages related to these experiments, there are experimental limitations, such as interspecies differences in lung physiology and species-specific reaction to protein-loaded nanoparticles [32].

## 8. Toxicity Concerns Regarding Protein-Loaded Nanoparticle Inhalation

Despite the benefic therapeutic effects of protein-loaded nanoparticles, it must be widely considered their possible toxicological effect as well. The assessment of exposure doses is useful to determine the doses at which the formulation is toxic and establish the therapeutic window intervals. In vitro, ex vivo and in vivo assays provide data on different parameters that contribute to a thorough knowledge of the formulation behavior [28].

The major concern about pulmonary administration of therapeutic proteins is the possibility of immunological reactions, in which the body may recognize them as antigens. In addition, when proteins are loaded into nanoparticles and administrated to the lungs, the safety of the excipients must be verified, both in short-term and long-term use [4]. Upon delivery into the lung, nanoparticles are absorbed and distributed in the body, potentially reaching several organs. These properties are highly attractive for pharmaceutical treatment and diagnosis of diseases, however, may also accumulate in the organs and pose potential toxicity [62,116]. The uptake of nanoparticles by cells has also been described in the literature, causing genotoxicity by accumulation of genetic alterations, DNA damage, or release of reactive oxygen species and proinflammatory cytokines [108,117]. Although the polymeric nanoparticles are biocompatible and biodegradable, their interaction with the immune system cells and red blood cells may cause hemotoxicity and cytotoxicity, inflammation, and oxidative stress [117]. Exacerbated inflammatory responses seem to be associated with particle size; the smaller show greater activation of the immune system compared to bigger particles. Brown et al. showed that 64 nm polystyrene nanoparticles significantly activated the IL-8 expression in A459 cells and neutrophil influx compared to 535 nm particles [118]. The authors attributed the cytotoxicity and proinflammatory effects to the large surface area of the smaller nanoparticles. Still, biodegradable polymeric nanoparticles seem to activate mild immune responses and inflammation. Dailey et al. developed diethylaminopropylamine (DEAPA) polyvinyl alcohol (PVA)-grafted-PLGA (DEAPA-PVA-g-PLGA) biodegradable nanoparticles ranging from 75 nm to 200 nm in size and compared the proinflammatory potential of these nanoparticles with polystyrene nanoparticles of similar size [119]. Results showed a lower inflammatory response for the biodegradable nanoparticles compared to the polystyrene nanoparticles.

## 9. Conclusions

The delivery of therapeutic proteins by the pulmonary route is equally attractive as it is challenging. The easy access to the lungs enables topical and systemic delivery of proteins, non-invasive and non-painful administration, and patient compliance are the gold standard of successful formulation development and administration. However, the delivery of therapeutic proteins is still ineffective due to their high molecular weight, mucociliary clearance, and enzymatic degradation, which makes their delivery challenging. To address these issues, polymeric nanoparticles have been suggested to encapsulate therapeutic proteins and manufactured by several spray-drying methods. The advantages of spray-drying include easy manufacture and control of formulation parameters, long-term stability, cost-effective, and protection of the loaded proteins. The literature is still scarce on the behavior of the loaded protein, and few studies suggest proteins maintain their structure after formulation, and consequently their bioactivity and bioavailability.

Overall, the formulation of inhalable protein-loaded polymeric nanoparticles by spray-drying is challenging in many ways, from its production to its delivery into the lungs. Therefore, the stability of the formulation, nanoparticle size and dry powder features need to be fully addressed to have an optimal and effective formulation. The development of new therapeutic inhalable systems for lung the delivery of proteins is expected to increase in the near future.

## Figures and Tables

**Table 1:** Advantages and disadvantages of protein loading into nanoparticles.

Advantages	Disadvantages
✓ Biocompatibility ✓ Enhanced bioavailability ✓ Biodegradability ✓ Increased biological barriers uptake ✓ High loading capacity ✓ Increased residence time of proteins in the body ✓ Increased protein half-life in bloodstream ✓ Protection from hydrolysis and enzymatic degradation ✓ Targeted delivery to specific tissues or cells ✓ Sustained protein delivery ✓ Decreased number of administrations ✓ Reduced toxicity	✕ Possible modifications in the structure upon formulation and delivery ✕ Possible loss of protein bioactivity upon formulation and delivery

**Table 2:** Advantages and drawbacks of spray-drying.

Advantages	Drawbacks
✓ Single-step, rapid and continuous process ✓ High reproducibility ✓ Easy scale-up ✓ High cost-effectiveness ✓ High versatility ✓ Good ability to control particle size and morphology ✓ High efficiency in encapsulation of drugs within polymeric carriers ✓ Allows manipulation of labile or heat-sensitive substances without significant degradation ✓ Good final product properties and quality ✓ Suitable for formulation of dry powders for inhalation	✕ Possible physical and chemical instability of therapeutic proteins ✕ Low yield at a lab scale ✕ Large evaporation rates due to high liquid feed requirement ✕ High pumping power

**Table 3:** Physicochemical properties of inhalable protein-loaded polymeric nanoparticles.

Carrier	Drug	Particle Size (nm)	Polydispersity Index	Zeta Potential (mV)	Drug Entrapment (%)	References
Glycol chitosan Np	Calcitonin	0.245	0.264	27.4 ± 4.1	54.2 ± 2.6	[88]
Glycol chitosan–thioglycolic acid Np	Calcitonin	0.332	0.337	22.3 ± 1.9	63.6 ± 5.9	[88]
PLGA Np	α-1 antitrypsin	100–1000	NA	NA	88–95	[89]
Chitosan Np	Insulin	289–404	NA	26–32	75–83	[15]
d,l-glyceraldehyde-poloxamer 188-coated Gelatin Np	Insulin	250–1010	0.276–0.387	−21 to −11	NA	[90]
Chitosan-coated PLGA Np	Pal-Ex4	695.7 ± 62.7	NA	28.5 ± 0.4	NA	[91]
Gelatin Np	BSA	187 ± 83	0.22 ± 0.007	−18.2 ± 2.61	NA	[10]
Chitosan Np	BSA	253 ± 110	0.28 ± 0.017	4.8 ± 1.08	NA	[10]
Alginate Np	BSA	556 ± 56	0.29 ± 0.014	−28.7 ± 0.89	NA	[10]
PLGA Np	BSA	160 ± 63	0.14 ± 0.017	−20.2 ± 1.16	NA	[10]
Chitosan-coated PLGA Np	BSA	191 ± 60	0.07 ± 0.006	−17.2 ± 1.34	NA	[10]
PLGA-PEG Np	BSA	335 ± 131	0.22 ± 0.033	−25.4 ± 1.01	NA	[10]
Chitosan-genipin Np	α-1 antitrypsin	30–100	NA	27.3 ± 2.5 (pH 4); 8.1 ± 3.1 (pH 7.4)	80–90	[92]
PLGA Np	Insulin	400	0.0691	NA	NA	[93]
Polybutylcyanoacrylate Np	Insulin	254.7	0.064	NA	79.1	[94]

NA: Not available. Np: Nanoparticles.

**Table 4:** In vitro lung epithelial cell culture models to evaluate protein-loaded polymeric nanoparticles performance.

Human Pulmonary Epithelial Cell Lines	
Cancer-Derived	Derived from Normal Lung
A549	16HBE14o-
Calu-1	BEAS-2B
Calu-3	9HTE16o-
Calu-6	1HAEo-
H441	CF/T43
HBE1	NuLi-1
A427	
HBE135-E6E7	

## Abbreviations and Acronyms

ALF	Artificial lysosomal fluid
BSA	Bovine serum albumin
cDNA	Complementary deoxyribonucleic acid
CF	5(6)-carboxyfluorescein
DEAPA	Diethylaminopropylamine
DEAPA-PVA-g-PLGA	Diethylaminopropylamine PVA-grafted-PLGA
DNA	Deoxyribonucleic acid
DPPC	Dipalmitoyl phosphatidylcholine
hAELVi	Human alveolar epithelial lentivirus
HBSS	Hanks balanced salt solution
IPL	Isolated perfused lung
MMAD	Mass median aerodynamic diameter
Pal-EX4	Palmitic acid-modified exendin-4
PBS	Phosphate-buffered saline
PCLS	Precision cut lung slices
PdI	Polydispersity index
PEG	Polyethylene glycol
PLGA	Polylactic-co-glycolic acid
PVA	Polyvinyl alcohol
SLF	Simulated lung fluids
YFP	Yellow fluorescein protein
ZP	Zeta potential
